# Binding of the Biogenic Polyamines to Deoxyribonucleic Acids of Varying Base Composition: Base Specificity and the Associated Energetics of the Interaction

**DOI:** 10.1371/journal.pone.0070510

**Published:** 2013-07-24

**Authors:** Ayesha Kabir, Gopinatha Suresh Kumar

**Affiliations:** Biophysical Chemistry Laboratory, Chemistry Division, CSIR-Indian Institute of Chemical Biology, Kolkata, India; University of Quebect at Trois-Rivieres, Canada

## Abstract

**Background:**

The thermodynamics of the base pair specificity of the binding of the polyamines spermine, spermidine, putrescine, and cadaverine with three genomic DNAs *Clostridium perfringens*, 27% GC, *Escherichia coli*, 50% GC and *Micrococcus lysodeikticus*, 72% GC have been studied using titration calorimetry and the data supplemented with melting studies, ethidium displacement and circular dichroism spectroscopy results.

**Methodology/Principal Findings:**

Isothermal titration calorimetry, differential scanning calorimetry, optical melting studies, ethidium displacement, circular dichroism spectroscopy are the various techniques employed to characterize the interaction of four polyamines, spermine, spermidine, putersine and cadaverine with the DNAs. Polyamines bound stronger with AT rich DNA compared to the GC rich DNA and the binding varied depending on the charge on the polyamine as spermine>spermidine >putrescine>cadaverine. Thermodynamics of the interaction revealed that the binding was entropy driven with small enthalpy contribution. The binding was influenced by salt concentration suggesting the contribution from electrostatic forces to the Gibbs energy of binding to be the dominant contributor. Each system studied exhibited enthalpy-entropy compensation. The negative heat capacity changes suggested a role for hydrophobic interactions which may arise due to the non polar interactions between DNA and polyamines.

**Conclusion/Significance:**

From a thermodynamic analysis, the AT base specificity of polyamines to DNAs has been elucidated for the first time and supplemented by structural studies.

## Introduction

**Figure 1:**
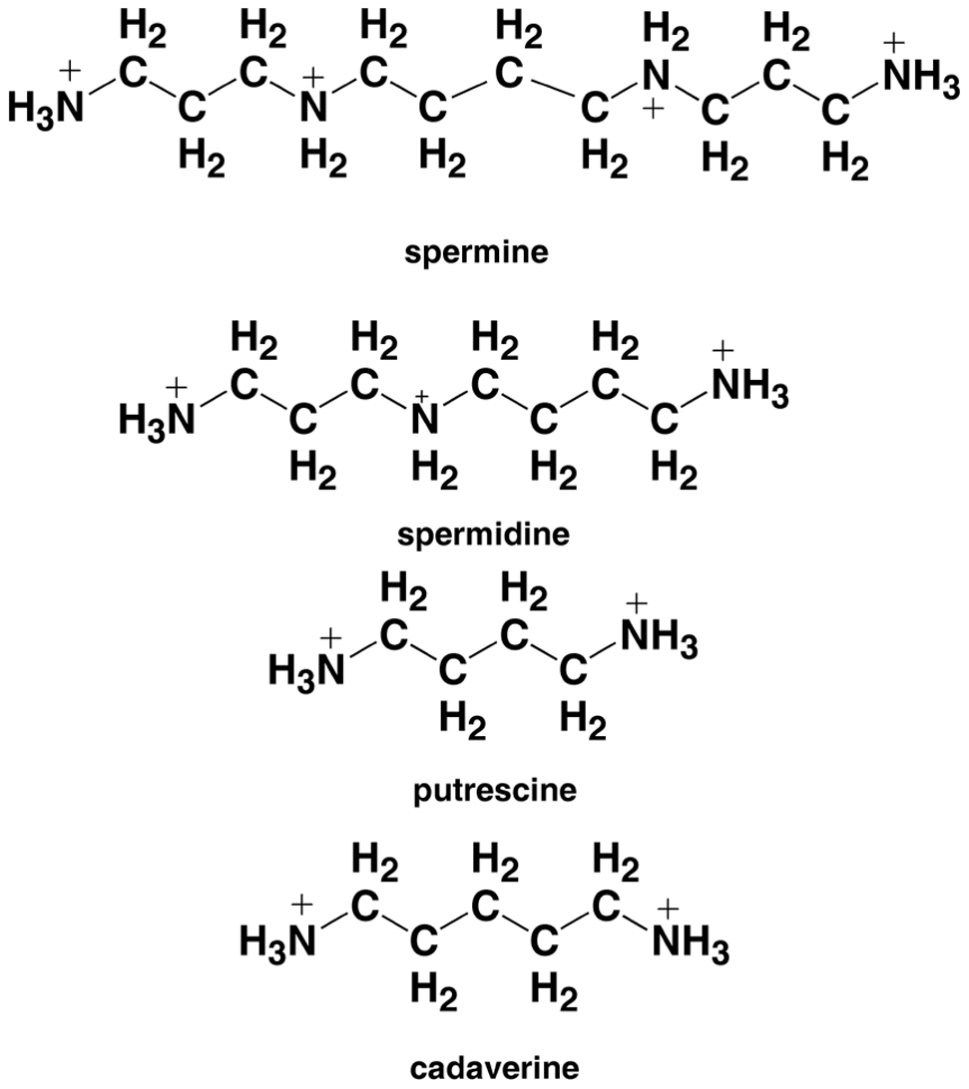
Chemical structure of polyamines.

Polyamines (Fig. 1) are cardinal indispensible molecules which sustain the structure, conformation, and function of nucleic acids and proteins thereby affecting cell growth and orchestrate cellular regulatory pathways and functions like gene regulation, DNA packaging, proliferation etc [1]–[6]. Numerous studies have shown that polyamines accumulate in cancer cells and a higher concentration of polyamines is usually correlated to cancerous growth. Studies have also demonstrated that a reduction of polyamine concentration usually has cytotoxic effects on cells through inhibition of growth or promotion of apoptosis in the cell. The effectiveness of polyamine analogs as antiproliferative agents against many tumor cell lines provides evidence for nucleic acid interaction [7]–[10]. Polyamines interaction with nucleic acids have also been shown to affect the stability of double and triple stranded DNA, protect DNA from oxidative stress, damaging agents, ionizing radiation, and endonuclease digestion etc [11]–[14].

Various studies have been performed in understanding the in vitro interaction of polyamines and derivatives with DNA, RNA and oligonucleotides [15]–[28]. However, the precise nature of the interaction of polyamines with DNA still remains obscure. Many of these studies have suggested electrostatic interaction to be the leading mode of interaction and both major and minor grooves have been proposed to be the target of polyamines. Also the base composition (and sequence) of the process of interaction between spermine and synthetic polynucleotides has shown significant differences between AT and GC base pairs [29]. Furthermore, recent studies divulge the involvement of both intra and interstrand interactions between polyamines and DNA, where the interstrand interaction would explain the ability of spermine and spermidine to offer higher protection [23]. X-ray crystallographic analyses indicated that polyamines occupy specific sites on non-B-DNA forms like A-DNA [30]–[32], Z-DNA [33], [34] and tRNA [35]. NMR studies have indicated the importance of the chemical nature of the nitrogens located in the polyamines in binding. These studies also contradict the simple notion that polyamines just behave as polycations with a hydrocarbon chain [17]. Thus, although a large amount of data is available on the structural aspects of polyamine DNA interactions, the thermodynamics of their binding to DNA is not yet elucidated except for a few reports [36], [37].

In this work we investigate the interaction of polyamines with genomic DNAs of varying base composition in order to understand the energetics of the base specificity of their interaction. We studied spermine, spermidine, putrescine and cadaverine with DNAs from low GC (27%) to high GC (72%) with the intension to characterize the base specificity of the interaction to correlate the structural and energetic aspects and to have a complete and unambiguous understanding of the interaction profile.

## Materials and Methods

### Biochemicals

DNA samples from *Clostridium perfringens* (CP) DNA (Type XII, 27% GC), *Eschereria coli* (EC) DNA (Type I, 50% GC) and *Micrococcus lysodeikticus* (ML) DNA (Type XI, 72% GC) were the products of Sigma-Aldrich Corporation (St. Louis, MO, USA). Spermine, spermidine, putrescine, cadaverine (all as hydrochloride salt) and ethidium bromide were purchased from Sigma-Aldrich.

### Preparation of Stock Solutions

The DNA was further purified by ethanol precipitation. The dried DNA sample was suspended in Citrate-Phosphate (CP) buffer and kept gently stirred overnight at 277.15 K. The clear solution was centrifuged and used for all the studies. The A_260_/A_280_ ratio value for all the three DNA samples were about 1.82, consistent with low protein content. The samples were sonicated to a uniform size of about 280±50 base pairs. The nativeness of the DNA samples was confirmed from optical melting and differential scanning calorimetry experiments where transitions with sharp melting temperatures were observed. The concentration of DNA was determined spectrophotometrically and expressed in terms of base pairs by using molar extinction coefficient values of 12,600, 13,200 and 14,800 M^−1^ cm^−1^, respectively, at 260 nm for CP, EC and ML DNAs. All experiments were done in citrate-phosphate (CP) buffer (20 mM Na^+^), pH 7.0, containing 10 mM Na_2_HPO_4_ and the pH was adjusted using citric acid except the ethidium bromide displacement assay. The buffer solutions were filtered through Millipore filters (Millipore, India Pvt. Ltd, Bangalore, India) of 0.22 µM to remove any particulate matter.

## Methods

### Isothermal Titration Calorimetry

ITC titrations were performed in a MicroCal VP-ITC unit (MicroCal, Inc., Northampton, MA, USA) at 293.15 K. Typically, 1.4235 mL of macromolecule (90–150 µM DNA base pair), loaded in the calorimetric cell was titrated against 600 µM polyamine solution using a 299 µL syringe, rotating at 254 r.p.m. Control experiments were performed by titrating polyamines to the buffer alone. The resulting thermograms were analyzed using single set of binding sites model of the MicroCal LLC software based on the Levenberg-Marquardt non-linear least squares curve fitting algorithm as described in details previously [38], [39], [40] to provide the binding affinity (*K_a_*), binding stoichiometry (N), and enthalpy of binding (Δ*H* ^o^). The binding free energy (Δ*G* ^o^) and the entropic contribution to the binding (*T*Δ*S* ^o^), were then calculated from standard thermodynamic relationships as described elsewhere [38], [41].

### Optical Thermal Melting Experiments

Melting profiles were registered on a Shimadzu Pharmaspec 1700 unit (Shimadzu Corporation, Kyoto, Japan) equipped with the Peltier controlled TMSPC-8 model accessory [42]. The sample solutions were prepared by mixing DNA (20 µM base pair) with varying concentrations of the polyamine under investigation in the degassed buffer. They were loaded into the eight cell micro optical cuvettes of 1 cm path length and heated at a rate of 1 K/min. The change in absorbance at 260 nm was continuously monitored until no further change occurred as revealed from the profiles. The melting temperature (*T_m_*) was taken as the midpoint of the melting transition as determined by the maxima of the first derivative plots.

### Differential Scanning Calorimetry

A Microcal VP-differential scanning calorimeter (DSC) (MicroCal Inc.,Northamption, USA) was employed to measure excess heat capacities as a function of temperature as described previously [39]. In DSC experiments at first both the sample and reference cells were filled with the buffer solution, equilibrated at 303.15 K for 15 minutes and scanned from 303.15 to 393.15 K at a scan rate of 60 K/hour. The buffer scans were repeated till reproducible results were obtained and on the cooling cycle, the sample cell was rinsed and loaded with DNA solution and then with the polyamine-DNA complexes of different D/P (polyamine/DNA base pair molar ratio) ratios and scanned. Each experiment was repeated twice with separate fillings. The DSC thermograms were analyzed using the Origin 7.0 software to determine the transition temperature (*T*_m_) and calorimetric transition enthalpy (Δ*H* ^o^_cal_) as described earlier [40], [41]. The calorimetric enthalpy is model-independent and unrelated to the nature of the transition. The *T*_m_ is defined as the temperature at which excess heat capacity is maximum. The model-dependent van’t Hoff enthalpy (Δ*H* ^o^_v_) was obtained by shape analysis of the calorimetric data and the cooperativity factor was obtained from the ratio of Δ*H* ^o^_cal_ and Δ*H* ^o^_v_. The reversibility of the transitions was also checked by allowing the sample to cool slowly (10 K/hour) to 303.15 K and then performing a repeat scan.

### Ethidium Bromide Displacement Assay

Steady state fluorescence measurements were performed either on a Shimadzu RF-5301PC fluorescence spectrometer (Shimadzu Corporation) or Perkin Elmer LS 55 fluorescence spectrometer (Perkin Elmer Inc. USA). Ethidium bromide displacement assay experiments were performed in 0.01 M SHE buffer, pH 7.2 [43]. The sample cuvette was thermostated at 293.15 K. The working solution (3 mL) contained 12 µM of DNA with 1.2 µM of ethidium bromide. This solution was kept stirred and the fluorescence emission spectrum was measured in the range 510–650 nm exciting at 490 nm. The fluorescence intensity at the wavelength maximum viz. 595 nm was noted. Concentrated polyamine solutions were added in small aliquots and fluorescence intensity at 595 nm recorded after each addition, stirring and equilibration till the fluorescence intensity quenched by 50%. To evaluate the nonspecific fluorescence decrease due to dilution factor, appropriate control experiments were performed by adding identical aliquots of buffer solution into the ethidium bromide–DNA complex. The polyamine concentration required to quench the fluorescence of the ethidium-DNA complex by 50% was derived from the plot of variation of the relative fluorescence intensity at 595 nm versus polyamine concentration.

### Circular Dichroism Studies

A Jasco model J815 spectropolarimeter (JASCO International Co., Hachioji, Japan) interfaced with a thermal programmer model 425 L/15 and controlled by the Jasco software was used for the circular dichroic measurements in rectangular strain free quartz cuvettes of 1 cm path length at 293.15±0.5 K [44]. Spectra presented here were obtained using a scan rate of 50 nm/min., a bandwidth of 1.0 nm and sensitivity of 100 milli degrees averaged from five successive accumulations and after subtraction of buffer baseline, followed by smoothening using the inbuilt Jasco software of the unit. The molar ellipticity [θ] values are expressed in terms of DNA base pair in the wavelength region of 200–400 nm. The calibration of the CD unit was routinely checked using an aqueous solution of d-10 ammonium camphor sulphonate.

## Results and Discussion

### Thermodynamics of the Interaction by Isothermal Titration Calorimetry

**Figure 2:**
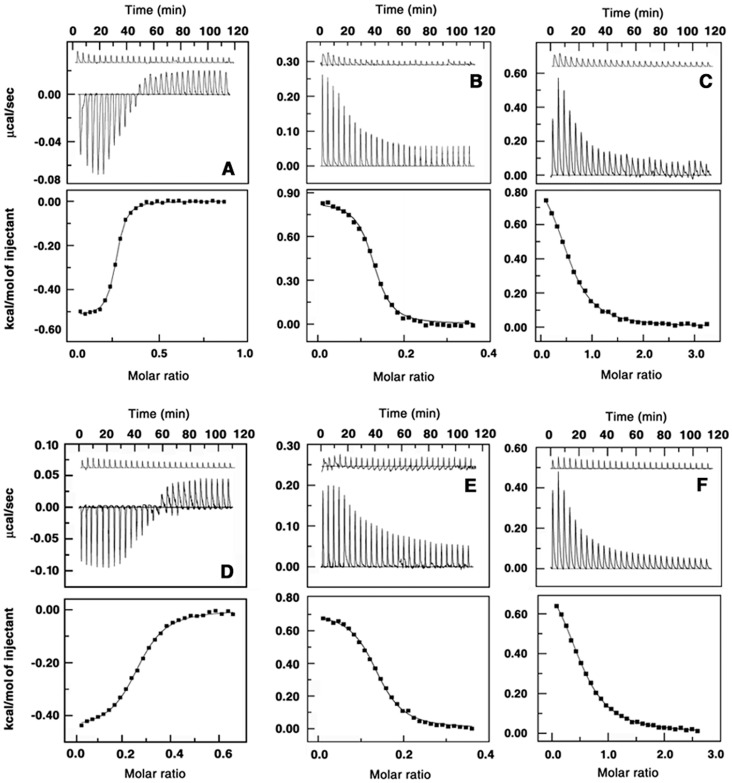
ITC profiles for the titration of polyamines with DNAs. Titration of spermine with (A) CP DNA (B) EC DNA (C) ML DNA and spermidine with (D) CP DNA (E) EC DNA (F) ML DNA at 293.15 K. The top panels represent the raw data for the sequential injection of polyamines into a solution of DNA and the bottom panels show the integrated heat data after correction of heat of dilution against molar ratio of DNA/[polyamine]. The data points were fitted to one site model and the solid line represent the best fit data.

The studies on the interaction of polyamines have been carried out with three genomic DNAs of varying base composition. Comparative analysis of the binding enables us to visualize the energetic scenario in terms of base composition. The ITC profiles for the binding of the four polyamines to the three DNAs are presented in Fig. 2. Each of the heat burst curve in the figure corresponds to a single injection of polyamine solution into the DNA solution that was corrected by the respective dilution heats derived from the control titration of identical amounts of polyamines into buffer alone. The resulting corrected heat plotted as a function of molar ratio is depicted in the lower panel where data points reflect the experimental injection heats while the solid lines reflect the calculated fits of the data. It can be seen that in each case there is only one binding event and the data were fitted to a model of single set of identical sites that yielded a fairly reasonable fitting of the experimental data. The titration of polyamines to CP DNA results in negative peaks which reveal the binding to be exothermic. On the other hand, for EC DNA and ML DNA, the profiles showed positive peaks in the plot of power versus time, revealing the binding to be endothermic. Also, it can be noted that the binding of the polyamines to ML DNA resulted in a higher positive enthalpy than that for the EC and CP DNA. The results obtained from the thermograms are summarized in Table 1, 2 and 3. The observations from Fig. 2 and from the Tables could be summed up as follows. Spermine binding to CP DNA showed the highest binding affinity, of the order of 10^6 ^M^−1^. With EC and ML DNAs the affinity of spermine was close and of the order of 10^5 ^M^−1^. For spermidine- CP DNA the affinity value was of the order of 10^5 ^M^−1^ while to EC and ML DNA the affinity was of the order of 10^4^ M^−1^. The binding of putrescine and cadaverine to CP DNA, EC DNA and ML DNA yielded a lower association constants in the rang 10^5^–10^4^ M^−1^. Thus, the preference of the polyamines to DNA vary in the order CP>EC>>ML DNA.

**Table 1:** ITC derived thermodynamic parameters for the binding of polyamines to CP DNA^a^.

Polyamines	Temperature (K)	*K_a_* (×10^5 ^M^−1^)	N	Δ*G* ^o^ (kcal/mol)	Δ*H* ^o^ (kcal/mol)	*T*Δ*S* ^o^ (kcal/mol)	Δ*C_p_* ^o^ (cal/mol K)
Spermine	283.15	36.7±0.56	0.271±0.002	−8.500±0.012	−0.175±0.002	8.325±0.010	−35.5±0.03
	288.15	29.6±0.32	0.260±0.001	−8.543±0.009	−0.360±0.003	8.183±0.025	
	293.15	18.5±0.10	0.227±0.001	−8.411±0.013	−0.529±0.003	7.882±0.015	
Spermidine	283.15	6.94±0.50	0.307±0.001	−7.582±0.014	−0.167±0.001	7.415±0.020	−28.6±0.05
	288.15	5.37±0.38	0.299±0.002	−7.548±0.011	−0.315±0.003	7.233±0.011	
	293.15	4.79±0.26	0.276±0.002	−7.602±0.019	−0.453±0.004	7.149±0.014	
Putrescine	283.15	2.14±0.16	0.330±0.002	−6.897±0.011	−0.133±0.009	6.764±0.009	−22.9±0.04
	288.15	1.74±0.18	0.329±0.007	−6.918±0.013	−0.236±0.004	6.682±0.013	
	293.15	1.18±0.07	0.244±0.002	−6.808±0.007	−0.362±0.008	6.446±0.014	
Cadaverine	283.15	1.09±0.12	0.273±0.003	−6.531±0.010	−0.132±0.002	6.399±0.020	−16.1±0.06
	288.15	0.87±0.09	0.213±0.002	−6.506±0.015	−0.228±0.007	6.278±0.011	
	293.15	0.69±0.09	0.184±0.003	−6.505±0.009	−0.293±0.012	6.212±0.008	

a All the data in this table are derived from ITC experiments conducted in 20 mM [Na^+^] citrate-phosphate buffer, pH 7.0 and are average of four determinations, *K_a_* and Δ*H* ^o^ values were determined from ITC profiles fitting to Origin 7 software as described in the text. The values of Δ*G* ^o^ and *T*Δ*S* ^o^ were determined using the equation Δ*G* ^o^ = −RTln*K_a_*, and *T*Δ*S* ^o^ = −Δ*H* ^o^−Δ*G* ^o^. All the ITC were fit to a model of single binding sites.

**Table 2:** ITC derived thermodynamic parameters for the binding of polyamines to EC DNA^a^.

Polyamines	Temperature (K)	*K_a_* (x10^5^ M^−1^)	N	Δ*G* ^o^ (kcal/mol)	Δ*H* ^o^ (kcal/mol)	*T*Δ*S* ^o^ (kcal/mol)	Δ*C_p_* ^o^ (cal/mol K)
Spermine	283.15	7.29±0.78	0.132±0.001	−7.604±0.023	1.141±0.006	8.745±0.015	−30.7±0.03
	288.15	5.83±0.74	0.130±0.001	−7.607±0.015	1.004±0.013	8.611±0.021	
	293.15	4.88±0.49	0.127±0.001	−7.634±0.014	0.834±0.009	8.468±0.011	
Spermidine	283.15	4.70±0.36	0.147±0.002	−7.358±0.011	0.962±0.050	8.320±0.023	−24.4±0.02
	288.15	3.47±0.27	0.144±0.001	−7.288±0.024	0.805±0.008	8.093±0.011	
	293.15	2.23±0.14	0.138±0.001	−7.165±0.012	0.717±0.007	7.882±0.016	
Putrescine	283.15	0.844±0.03	0.178±0.006	−6.364±0.021	0.796±0.007	7.160±0.013	−14.1±0.04
	288.15	0.626±0.03	0.178±0.002	−6.324±0.006	0.703±0.005	7.027±0.004	
	293.15	0.468±0.02	0.146±0.002	−6.260±0.010	0.655±0.012	6.915±0.008	
Cadaverine	283.15	0.725±0.02	0.206±0.001	−6.302±0.009	0.745±0.005	7.047±0.018	−14.0±0.05
	288.15	0.574±0.02	0.191±0.001	−6.281±0.013	0.631±0.006	6.912±0.011	
	293.15	0.357±0.01	0.192±0.002	−6.111±0.011	0.599±0.008	6.710±0.017	

a All the data in this table are derived from ITC experiments conducted in 20 mM [Na^+^] citrate−phosphate buffer, pH 7.0 and are average of four determinations, *K_a_* and Δ*H* ^o^ values were determined from ITC profiles fitting to Origin 7 software as described in the text. The values of ΔG^o^ and *T*Δ*S* ^o^ were determined using the equation Δ*G* ^o^ = −RTln*K_a_*, and *T*Δ*S* ^o^ = Δ*H* ^o^−Δ*G* ^o^. All the ITC were fit to a model of single binding sites.

**Table 3:** ITC derived thermodynamic parameters for the binding of polyamines to MLDNA^a^.

Polyamines	Temperature (K)	*K_a_* (×10^4 ^M^−1^)	N	Δ*G* ^o^ (kcal/mol)	Δ*H* ^o^ (kcal/mol)	*T*Δ*S* ^o^ (kcal/mol)	Δ*C_p_* ^o^ (cal/mol K)
Spermine	283.15	6.52±0.02	0.714±0.004	−6.230±0.012	1.217±0.009	7.447±0.023	−24.5±0.02
	288.15	5.20±0.09	0.682±0.009	−6.223±0.021	1.096±0.011	7.319±0.015	
	293.15	3.33±0.15	0.551±0.004	−6.060±0.013	0.972±0.012	7.032±0.013	
Spermidine	283.15	4.55±0.03	0.646±0.011	−6.034±0.011	1.101±0.015	7.135±0.018	−21.2±0.01
	288.15	2.88±0.06	0.607±0.021	−5.890±0.013	0.997±0.012	6.887±0.015	
	293.15	2.20±0.02	0.536±0.005	−5.821±0.020	0.889±0.009	6.710±0.019	
Putrescine	283.15	3.49±0.01	0.617±0.004	−5.892±0.021	1.017±0.021	6.909±0.014	−15.1±0.05
	288.15	2.84±0.03	0.572±0.009	−5.877±0.010	0.952±0.019	6.829±0.011	
	293.15	2.04±0.01	0.534±0.007	−5.781±0.009	0.866±0.014	6.651±0.007	
Cadaverine	283.15	3.79±0.02	0.727±0.017	−5.923±0.012	1.014±0.013	6.937±0.011	−13.7±0.03
	288.15	2.87±0.02	0.595±0.017	−5.889±0.018	0.940±0.030	6.829±0.016	
	293.15	2.02±0.06	0.531±0.015	−5.716±0.021	0.877±0.008	6.593±0.011	

a All the data in this table are derived from ITC experiments conducted in 20 mM [Na^+^] citrate-phosphate buffer, pH 7.0 and are average of four determinations, *K_a_* and Δ*H* ^o^ values were determined from ITC profiles fitting to Origin 7 software as described in the text. The values of Δ*G* ^o^ and *T*Δ*S* ^o^ were determined using the equation Δ*G* ^o^ = −RTln*K_a_*, and *T*Δ*S* ^o^ = Δ*H* ^o^−Δ*G* ^o^. All the ITC were fit to a model of single binding sites.

The binding of the polyamines to CP DNA was driven by large positive entropy changes and relatively small negative enthalpy changes whereas for EC and ML DNA the interactions were clearly entropy driven. The strong positive entropy term is suggestive of the disruption and release of water molecules on interaction and the same is more in the AT rich CP DNA. It is known that the AT base pairs are associated with more water of hydration than the GC base pairs. The free energy changes for binding spermine and spermidine to CP DNA, EC DNA and ML DNA were in the range −8.5 to −7.6 kcal/mol, −7.6 to −7.2 kcal/mol and −6.2 to −5.8 kcal/mol, respectively. Thus, ITC data suggests an AT specificity for the binding of the polyamines to the DNA and confirms the strength of interaction as spermine>spermidine>putrescine >cadaverine.

### Temperature Dependence of the Binding: Heat Capacity Changes

The constant pressure heat capacity change (Δ*C_p_* ^o^) of polyamine-DNA interactions were determined from the temperature dependence of the binding enthalpy employing the standard relationship, Δ*Cp* ^o = ^[δΔ*H* ^o^/δT]_p_. This information may provide valuable insights into the type and magnitude of forces involved in the interaction. Temperature dependent ITC experiments were conducted at three temperatures viz. 283.15, 288.15, 293.15 K. The thermograms showed only binding event at all these temperatures. The thermodynamic parameters evaluated at these temperatures are depicted in Table 1, 2, 3. It can be seen that as the temperature was increased, there was a decrease in the association constant of the binding while there was only a subtle change in the number of binding sites. Interestingly, there were remarkable changes in the enthalpy and entropy contributions. For CP DNA, with increasing temperature, the Δ*H* ^o^ values increased, but the *T*Δ*S* ^o^ values decreased in such a way so that the free energy change was minimal (Table 1). In the case of EC and ML DNAs, the Δ*H* ^o^ and values *T*Δ*S* ^o^ both decreased, but in a way such that the free energy change was minimum (Table 2 and 3, respectively).

**Figure 3:**
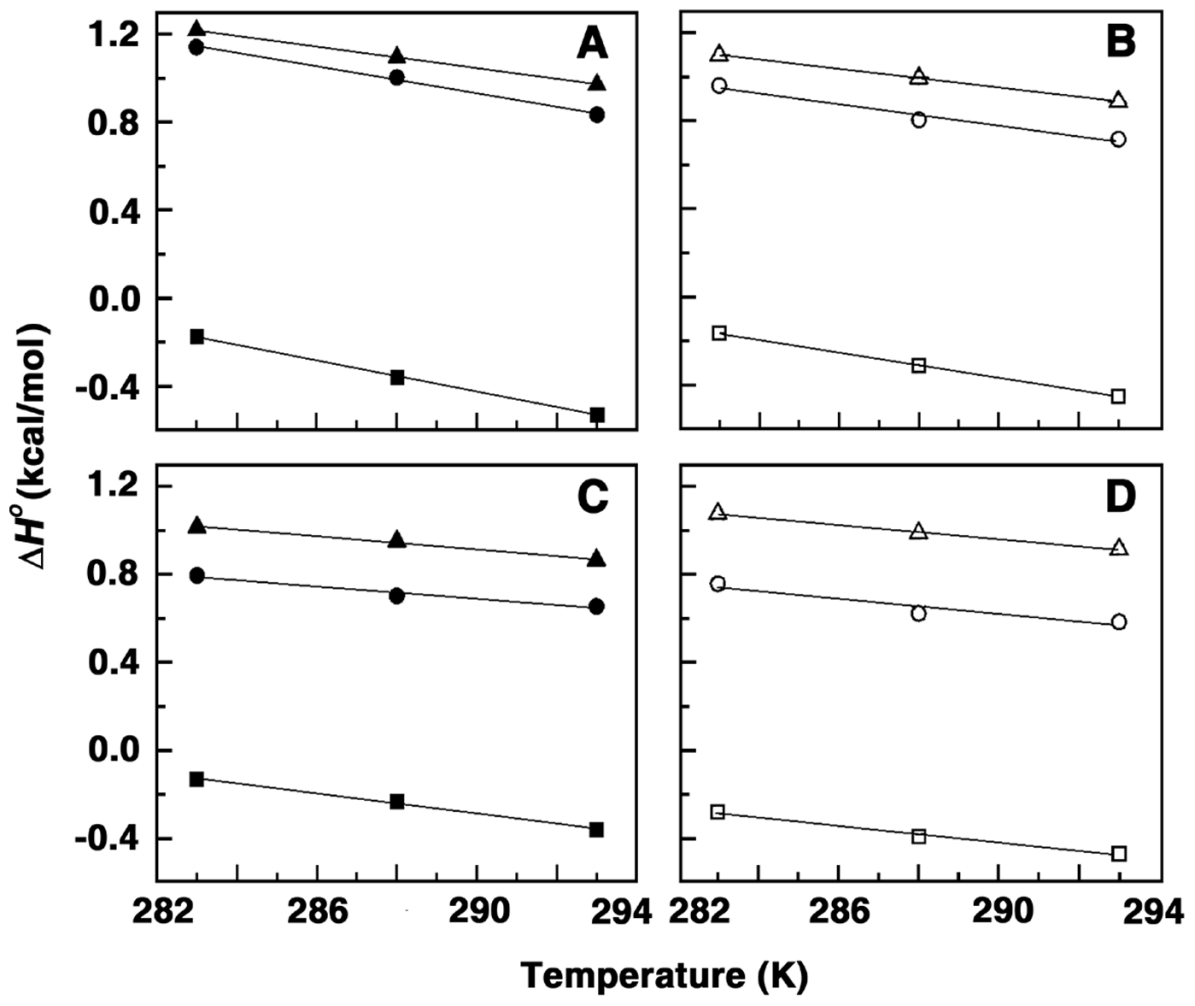
Plot of variation of enthalpy of binding (Δ*H^O^*) with temperature. Plots for the binding of (A) spermine with CP DNA (▪), EC DNA (•), ML DNA (▴), (B) spermidine with CP DNA (□), EC DNA (○), ML DNA (▵), (C) putrescine with CP DNA (▪), EC DNA (•), ML DNA (▴) and (D) cadaverine with CP DNA (□), EC DNA (○), ML DNA (▵).

A plot of the variation of Δ*H* ^o^ with temperature is presented in Fig. 3. The slopes of the lines for spermine, spermidine, putrescine, cadaverine revealed values of −35.5, −28.6, −22.9, −16.1 cal/mol.K for CP DNA, −30.7, −24.4, −14.1, −14.0 cal/mol.K for EC DNA, −24.5, −21.2, −15.1, −13.7 cal/mol.K for ML DNAs, respectively (Table 1, 2,3). It may be noted that negative heat capacity values are the hall mark of a variety of small molecules binding to DNA and RNA [45]–[49]. Usually observed large, negative standard heat capacity change are characteristic for specific changes in hydrophobic or polar group hydration and is considered as indicator of a dominant hydrophobic effect in the binding process. The changes in solvent accessible surface area have also been shown to be a large component of Δ*C_p_* ^o^ [50], [51], [52]. Here, the values of Δ*C_p_* ^o^ are non-zero indicating temperature dependence of the enthalpy change which suggests that the binding enthalpy becomes more favourable and binding entropy less favourable at higher temperatures. The values Δ*C_p_* ^o^ obtained here are lower than that was generally observed for DNA and RNA intercalators and hence supports the suggestion of a groove binding model with significant hydrophobic contribution to the binding. It is known that for DNA, change in structured water the minor groove can be associated with large heat capacity changes and release of such water of hydration is usually associated with the transfer of some non polar groups on binding in the grooves of the helix. Furthermore nonspecific binding by small molecules like what is seen here usually has much smaller Δ*C_p_* ^o^ changes. Four types or modes of DNA recognition by small molecules–sequence specific, nonspecific, minimal sequence specific and structure specific–have been discussed by Murphy and Churchill [53]. Small negative Δ*C_p_* ^o^ values are considered to be associated with a minimal sequence specific binding. Therefore, the slightly negative non-zero Δ*C_p_* ^o^ value that is observed for polyamine-DNA complexes appears to denote the structure specific binding. The higher values of Δ*C_p_* ^o^ for the binding of the polyamines to the AT rich CP DNA compared to others may suggest conformational differences in the DNA structures and also differences in the disruption of the water structure around the DNA base pairs on complex formation. It is known that AT base pairs have more water of hydration as compared to GC base pairs and the differences in the release of structured water consequent to the transfer of the polyamine molecules into the interior of the groove of the helix are higher for the AT rich DNA.

As mentioned above, for DNA and RNA intercalators a large hydrophobic contribution to the binding free energy may come from their aromatic ring system and binding may be energetically favourable [54]. From the relationship [55], Δ*G* ^o^ *_hyd_* = (80±10) × Δ*C_p_* ^o^ the free energy component for the hydrophobic transfer step of binding of these molecules may be evaluated. Extending this to DNA-polyamine system the Δ*G* ^o^ *_hyd_* values for binding of these polyamines spermine, spermidine, putrescine and cadaverine to CP DNA were deduced to be −2.84, −2.29, −1.83 and −1.29 kcal/mol, for EC DNA −2.46, −1.95, −1.13, −1.12 kcal/mol, and for ML DNA −1.96, −1.70, −1.21, −1.10 kcal/mol, respectively.

Although electrostatic interaction plays the major role in the interaction of polyamines to DNA, these results suggest significant importance for hydrophobic interactions as well. Also the Δ*G* ^o^ *_hyd_* is higher for CP DNA as compared to the ML DNA due to the higher AT base pair content and the higher water of hydration associated with the AT sequences. Together we can infer that the binding of polyamines to DNA duplexes does reflect AT specificity of binding.

### Enthalpy–entropy Compensation

**Figure 4:**
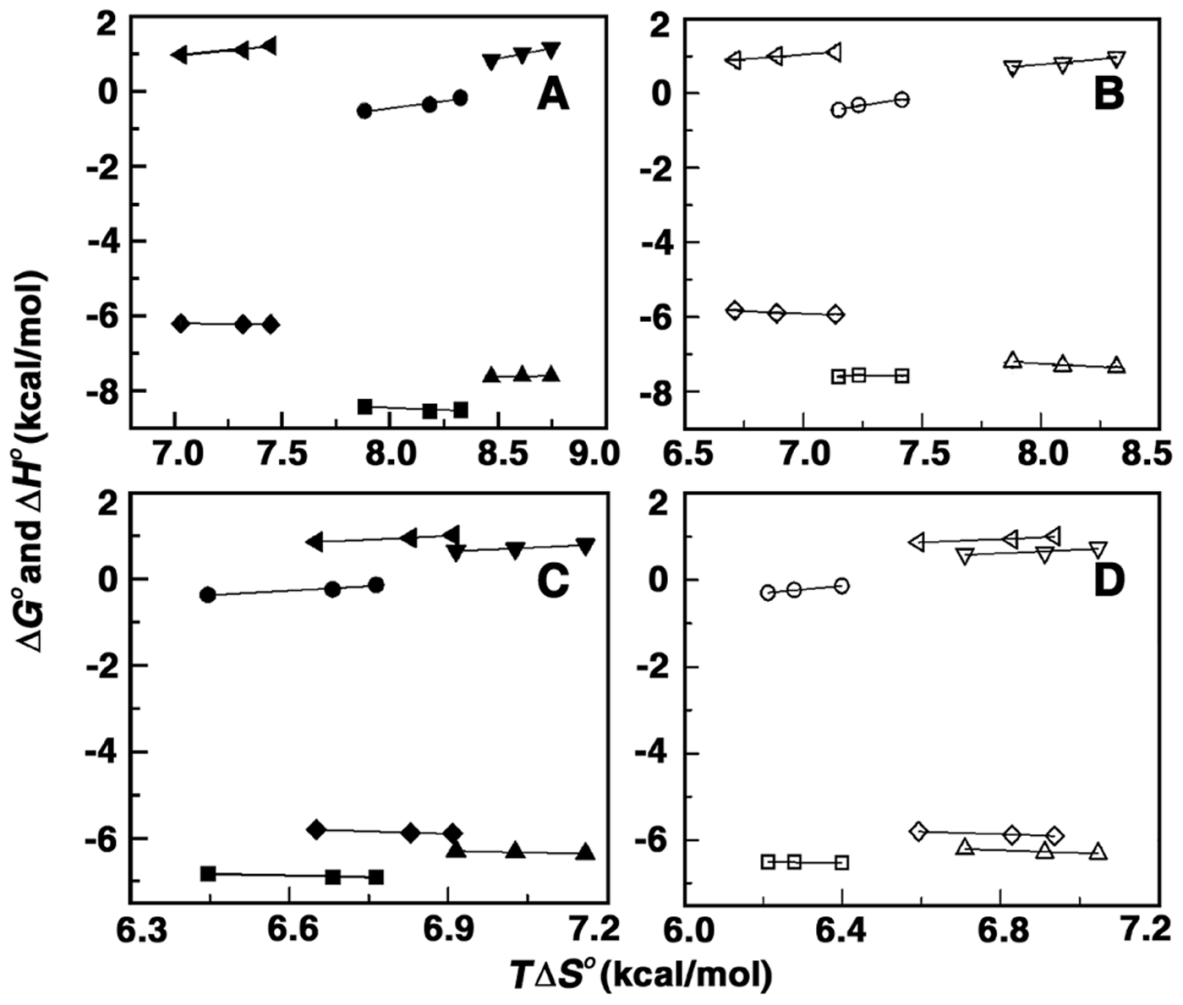
Plots of variationof thermodynamic parameters with entropy contribution. Plot of Δ*G^O^* and Δ*H^O^* versus *T*Δ*S^O^* for the binding of (A) spermine with CP DNA (▪,•), EC DNA (▴,▾), ML DNA (♦,◂) (B) Plot of Δ*G^O^* and Δ*H^O^* versus *T*Δ*S^O^* of spermidine with CP DNA (□,○), CT DNA (▵,▿), ML DNA (⋄,⊲), (C) Plot of Δ*G^O^* and Δ*H^O^* versus *T*Δ*S^O^* of putrescine with CP DNA (▪,•), EC DNA (▴,▾), ML DNA (♦,◂) and (D) Plot of Δ*G^O^* and Δ*H^O^* versus *T*Δ*S^O^* of cadaverine with CP DNA (□,○), EC DNA (▵,▿), ML DNA (⋄,⊲).

For polyamines binding to CP DNA with increase in temperature the binding enthalpy increased and the entropy term *T*Δ*S* ^o^ (a favourable term in the Δ*G* ^o^) decreased. The free energy exhibited only small changes in each case (Table 1). Similarly for EC DNA and ML DNA (endothermic reactions) the binding enthalpy and the entropy term decreased with increasing temperature. Here also the free energy change exhibited only small changes (Table 2, 3). Both the reaction enthalpy and entropy that are strong functions of temperature compensated each other to make the reaction free energy more or less independent of temperature. Many biomolecular interactions have been reported to exhibit this enthalpy-entropy compensation behaviour [56], [57] which suggests a significant hydrophobic component to the binding energies linked to the solvent reorganization accompanying binding interactions. Enthalpy change with *T*Δ*S* ^o^ shows a linear relationship with a slope near unity which is an indication of complete compensation. This occurs generally with Δ*Cp* ^o^ not equal to zero and Δ*Cp* ^o^>Δ*S* ^o^. In Fig. 4 the variation of Δ*H* ^o^ as a function of *T*Δ*S* ^o^ is presented for spermine and spermidine binding to the three DNAs. The values of the slope were 0.98, 0.99 and 1.09 for spermine and 0.98, 1.08 and 0.94 for spermidine binding to CP, EC and ML DNAs.

### Salt Dependence of the Calorimetric Data

The interaction of spermine, the polyamine that exhibited strongest binding with the DNAs was investigated at three [Na^+^] conditions viz. 10, 20 and 50 mM. Based on the trend here, the data could be generalized for polyamine-DNA binding. The data was analyzed in conjunction with van’t Hoff analysis that revealed the parsing of free energy in the binding of these polyamines. The relation between binding constant (*K*_a_) and [Na^+^] ion concentration has been described previously by Record et al [58]

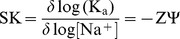
(1)where SK is equivalent to the number of counter ions released upon binding of a small molecule, Z is the apparent charge of the bound ligand and ψ is the fraction of sodium ions bound per DNA phosphate group.

**Figure 5:**
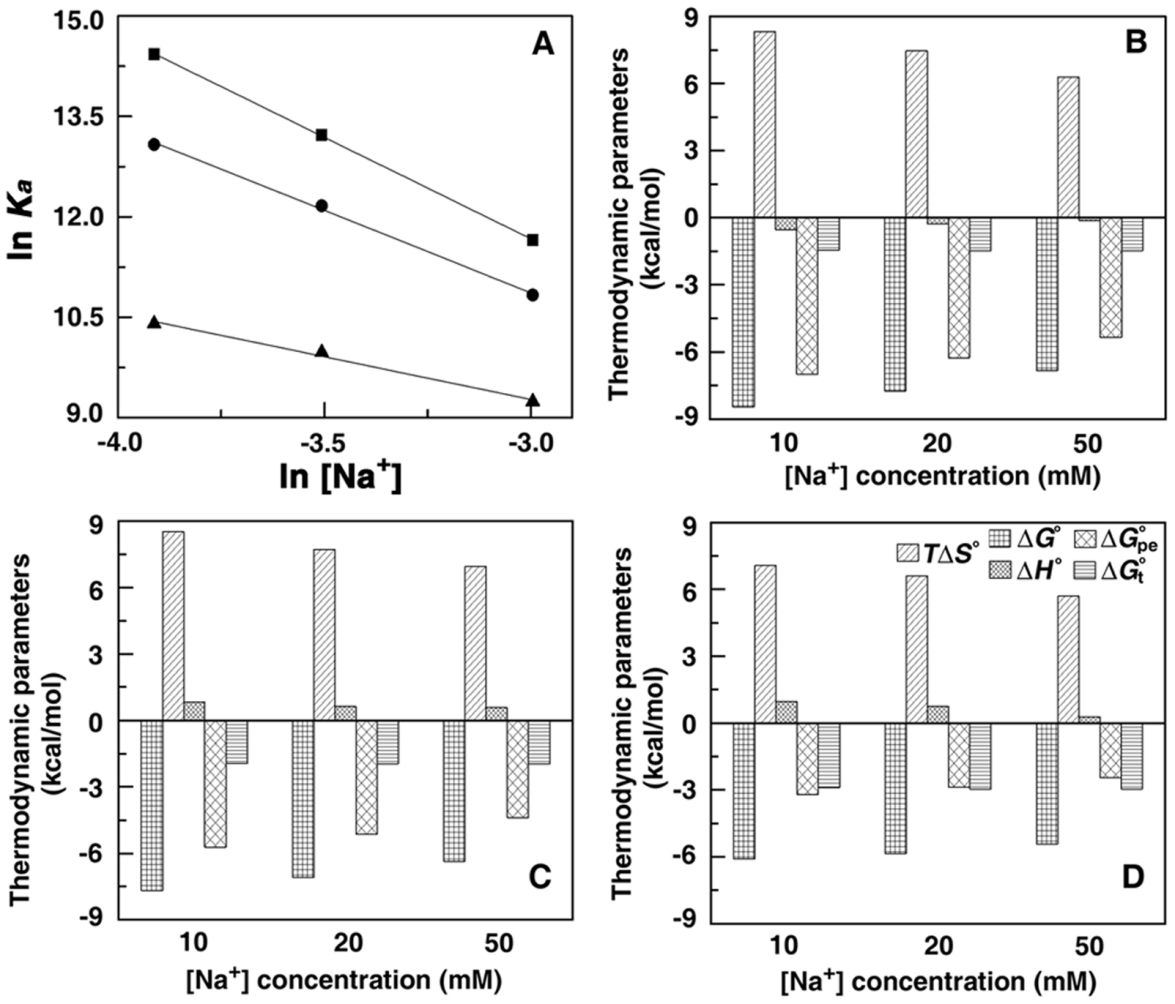
Plots of variation of salt dependent thermodynamic parameters. (A) Plot of ln *K_a_* versus ln [Na^+^] for the binding of spermine with CP DNA (▪), EC DNA (•), MLDNA (▴). Bar diagram describing the variation of magnitude of thermodynamics parameters at three salt concentrations for (B) CP DNA, (C) EC DNA and (D) ML DNA at 293.15 K.

As the salt concentration was enhanced the binding affinity decreased. The number of binding sites, however, varied only marginally. A plot of ln *K*_a_ versus ln [Na^+^] was linear and gave values of slope as ^_^3.03, −2.5 and ^_^1.28, respectively, with CP DNA, EC DNA and ML DNA (Fig. 5). The higher values of slope compared to the theoretical value are obtained due to the presence of multiple charges on spermine. Also these high values suggest strong electrostatic contacts of spermine with the DNA duplexes.

The observed free energies of the interaction are in the range −8.5 to −6.8 kcal/mol, −7.7 to −6.4 kcal/mol, −6.1 to −5.4 kcal/mol for CP, EC and ML DNA, respectively (Table 4).The observed free energy can be partitioned between the polyelectrolytic (Δ*G* ^o^_pe_) and non-polyelectrolytic (Δ*G* ^o^_t_) contributions which can be derived from the dependence of *K_a_* on [Na^+^]. The contribution to the free energy from the electrostatic interaction (polyelectrolytic) can be quantitatively determined from the relationship
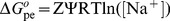
(2)where Zψ is the slope of the van’t Hoff plot. The Δ*G* ^o^_pe_ contributions at 20 mM [Na^+^] concentration have been determined to be around −6.99, −5.73, −3.21 kcal/mol, respectively, for spermine binding to CP, EC and ML DNA (Table 4) which are about 82, 74 and 53% of the total free energy of CP, EC and ML DNA (Fig. 5). These values reveal significant level of electrostatic contribution to the binding in each case. On the other hand, at 50 mM [Na^+^], there was significant reduction in the Δ*G* ^o^_pe_ values (Table 4). Therefore, we can state that our results are in accordance with a strong salt dependent interaction of polyamines with DNA.

**Table 4:** ITC derived thermodynamic parameters for the binding of spermine to CP, EC and ML DNA at 293.15 K at different [Na^+^] concentration^a^.

SPERMINE with	[Na^+^] mM	*K_a_* (×10^5 ^M^−1^)	N	Δ*G* ^o^ (kcal/mol)	Δ*H* ^o^ (kcal/mol)	*T*Δ*S* ^o^ (kcal/mol)	Δ*G* ^o^ *_pe_* (kcal/mol)	Δ*G* ^o^ *_t_* (kcal/mol)
	20	18.5±0.10	0.227±0.001	−8.456±0.006	−0.529±0.003	8.319±0.015	−6.992±0.012	−1.464±0.004
CP DNA	30	5.50±0.35	0.182±0.002	−7.746±0.011	−0.284±0.004	7.462±0.012	−6.267±0.013	−1.478±0.018
	50	1.15±0.09	0.130±0.005	−6.828±0.009	−0.137±0.006	6.299±0.009	−5.354±0.019	−1.474±0.010
	20	4.88±0.49	0.127±0.001	−7.678±0.012	0.837±0.009	8.515±0.013	−5.731±0.012	−1.947±0.010
EC DNA	30	1.81±0.25	0.122±0.004	−7.091±0.011	0.624±0.025	7.715±0.023	−5.137±0.006	−1.954±0.009
	50	0.51±0.02	0.100±0.001	−6.362±0.009	0.579±0.031	6.941±0.015	−4.388±0.009	−1.973±0.005
	20	0.33±0.02	0.551±0.007	−6.097±0.015	0.972±0.017	6.098±0.011	−3.209±0.007	−2.887±0.017
ML DNA	30	0.21±0.04	0.421±0.009	−5.846±0.018	0.756±0.049	5.847±0.005	−2.877±0.016	−2.969±0.004
	50	0.11±0.01	0.215±0.018	−5.415±0.008	0.271±0.029	5.415±0.020	−2.458±0.019	−2.957±0.013

a All data in this table are derived from ITC experiments conducted in citrate-phosphate buffer pH 7.0, and are an average of four determinations. *K_a_* and Δ*H* ^o^ values were determined from ITC profiles fitting to Origin 7 software as described in text. The values of Δ*G* ^o^ and *T*Δ*S* ^o^ were determined using the equations Δ*G* ^o^ = −*RT*ln*K_a_*, and *T*Δ*S* ^o^ *= *Δ*H* ^o^ *−*Δ*G* ^o^. All ITC profiles were fit to a model of single binding site.

### Stabilization of DNA by Polyamines: Optical Melting and DSC Studies

**Figure 6:**
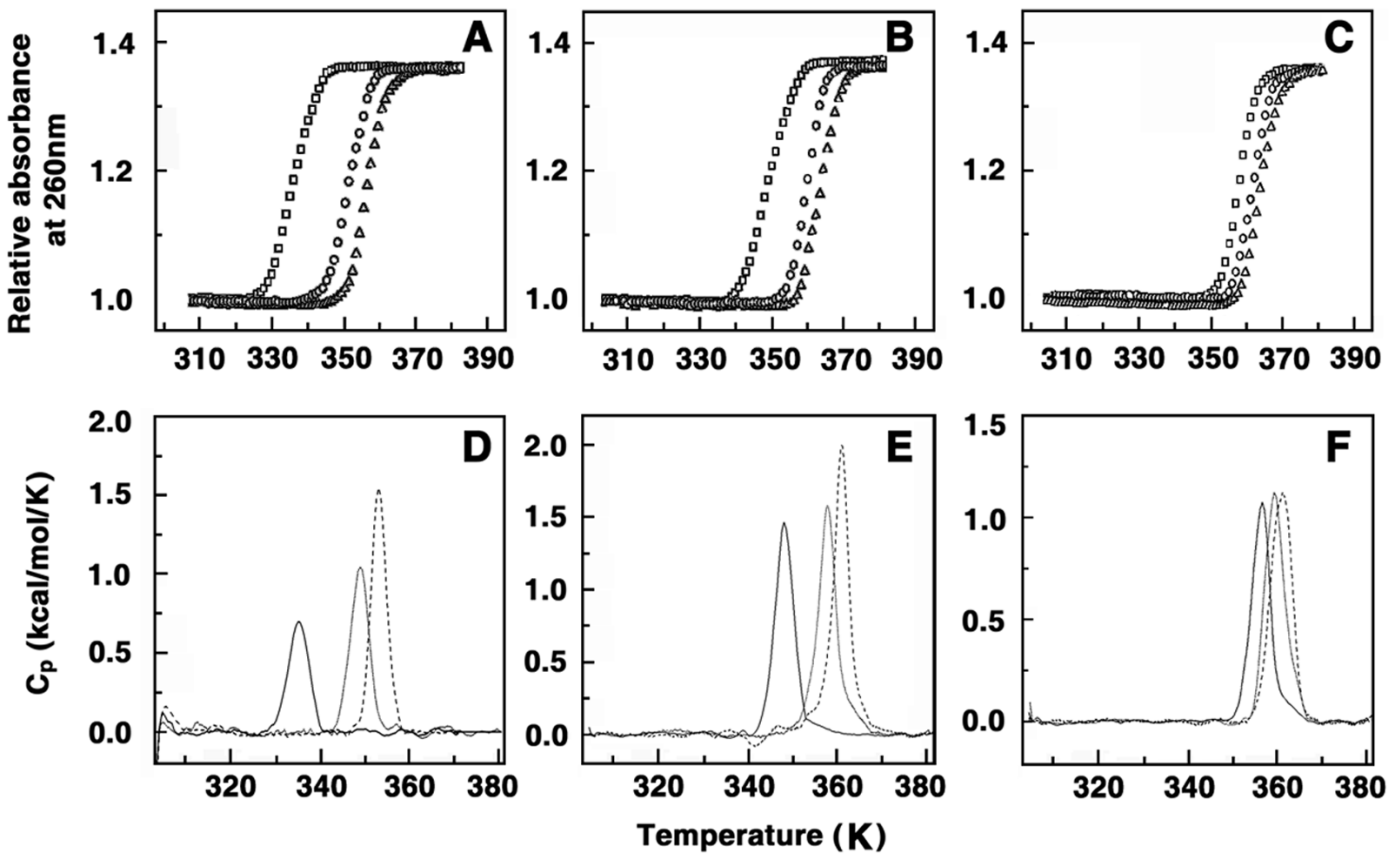
Melting profiles of DNA and DNA polyamine complexes. Optical melting profiles (upper panels) of (A) CP DNA (□), spermine-CP DNA(▵), spermidine-CP DNA (O), (B) EC DNA (□), spermine-EC DNA(▵), spermidine-EC DNA(O), (C) ML DNA (□), spermine-ML DNA(▵), spermidine-ML DNA(O). DSC melting profiles (lower panels) of (D) CP DNA (solid lines) (E) EC DNA (solid lines), (F) ML DNA (solid lines) and respective DNA–spermine complex (- - - -) and DNA-spermidine complex (….).

The melting profiles CP DNA, EC DNA and ML DNA and their complexes with the polyamines are presented in Fig. 6. CP, EC and ML DNA show sigmoidal melting curves with about 40% hyperchromicity and melting temperature of 335.96 K, 349.40 K and 357.28 K, respectively. In presence of the polyamines the stability of the DNAs enhanced as revealed by the enhancement of the melting temperature (*T_m_*). The *T_m_* is a characteristic of each DNA that is largely determined by the base composition of the DNA. Higher the percentage of G:C base pairs in the DNA the higher the melting temperature. Natural DNA may have different regions of A:T and G:C variations but once the strand starts melting at a position after that it’s a simple unzipping process of the helix irrespective of the base composition. Thus, a DNA with higher A:T content will require a lower threshold energy and thus a lower temperature to start unzipping, as compared to a DNA with higher G:C content. Under saturating conditions, the maximum Δ*T_m_* values (*T_m_* of the complex minus the *T_m_* of the DNA alone) obtained for spermine were 18.96 K, 12.97 K and 4.99 K, and with spermidine were 14.09 K, 9.77 K and 3.67 K with CP, EC and ML DNA (Table 5). Spermine and spermidine have greater stabilization effect than putrescine and cadaverine on all the three DNAs. There were no changes in either the cooperativity of the transition or the hyperchromicity in any of the systems.

**Table 5:** Thermal melting data and the binding constants from melting data at saturating concentrations of polyamines with CP DNA, EC DNA and ML DNA^a^.

Sample	Δ*T*_m_ (K)	Δ*H* ^o^ (kcal/mol)	Δ*H* ^o^_v_ (kcal/mol)	*K_T_*_m_ b×10^5^ (M^−1^)	*K*_obs_ c×10^5^ (M^−1^)
**CP DNA**	−	4.199±0.19	153.9±0.88	−	−
Spermine	18.96±0.01	6.989±0.19	224.4±0.78	11.92±0.21	10.23±0.16
Spermidine	14.09±0.01	5.694±0.09	182.3±0.38	3.98±0.16	3.51±0.03
Putrescine	8.15±0.02	5.496±0.05	148.7±0.17	1.15±0.01	1.05±0.02
Cadaverine	6.24±0.01	4.855±0.21	149.8±0.08	0.75±0.02	0.70±0.04
**EC DNA**	−	8.035±0.05	175.6±1.15	−	−
Spermine	12.97±0.01	9.267±0.03	212.3±0.85	9.01±0.02	6.70±0.01
Spermidine	9.77±0.01	8.510±0.02	180.7±0.55	4.80±0.01	3.80±0.02
Putrescine	4.70±0.02	7.948±0.09	183.0±2.59	0.76±0.01	0.62±0.01
Cadaverine	4.30±0.01	7.883±0.08	174.4±2.26	0.53±0.01	0.44±0.01
**MLDNA**	−	5.889±0.04	184.5±1.74	−	−
Spermine	4.99±0.01	7.039±0.02	173.5±0.55	0.53±0.02	0.38±0.01
Spermidine	3.67±0.02	6.590±0.06	175.6±1.88	0.26±0.01	0.19±0.01
Putrescine	3.29±0.01	6.033±0.06	213.7±2.50	0.22±0.01	0.16±0.01
Cadaverine	3.07±0.01	5.451±0.11	184.2±4.83	0.21±0.01	0.16±0.01

a Melting stabilization of DNA (Δ*T*_m_) in the presence of saturating amounts of polyamines are average of optical melting and DSC data.

b *K_T_*_m_ is the binding constant at the melting temperature.

c *K*_obs_ is the polyamine binding constant at 293.15 K determined using equations described in the text.

Thus we can infer that the effect on the DNA melting temperature increases as positively charged secondary amino group(s), gets longer. Also for spermine and spermidine the higher stabilization may be due to crosslink formation through electrostatic interaction bridging the groove of the helix, which makes the DNA more stable to heat denaturing effect [59]. These results clearly suggest higher stabilization of the DNAs of high AT content and as spermine>spermidine >putrescine>cadaverine corroborating the results from ITC and CD experiments.

The differential scanning calorimetric profiles (Fig. 6) also revealed *T_m_* values very close to that obtained from optical absorption melting experiments. In presence of saturating concentrations of the polyamines the melting temperatures of the CP, EC and ML DNA increased stabilizing them by Δ*T_m_* values of 18.96 K, 12.97 K, 4.99 K for spermine, 14.09 K, 9.77 K, 3.67 K for spermidine, 8.15 K, 4.70 K, 3.29 K for putrescine, 6.24 K, 4.30 K, 3.07 K for cadaverine (Table 5).

The above described *T_m_* results, however, may not be directly correlated with binding affinity although a higher Δ*T_m_* is tempting to suggest stronger binding affinity. To relate melting data with binding strength we calculated the binding constants of these molecules to the DNA duplex using the equation derived by Crothers [60]

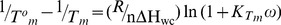
(3)where *T^o^_m_* is the optical melting temperature of the DNA in the absence of the drug, *T_m_* is the melting temperature in presence of saturating amounts of the drug, Δ*H_wc_* is the enthalpy of DNA melting obtained from the DSC experiment, R is the gas constant, (1.987216 cal. K^−1 ^mol^−1^), *K_Tm_* is the drug binding constant at the *T_m_*_,_ ω is the free drug activity that may be estimated by one half of the total drug concentration, and n is the site size of the drug binding. The calculated apparent binding constant at the melting temperature can be extrapolated to a reference temperature (say 293.15 K) using the standard relationship,
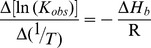
(4)where K_obs_ is the drug binding constant at the reference temperature T (in Kelvin) and Δ*H_b_*, the binding enthalpy which is determined from the isothermal titration calorimetry experiment (*vide infra*). The binding constants (* K* *_obs_*) calculated from the melting data using the above equations for 293.15 K are presented in (Table 5). These values represent the overall binding affinity that was higher for spermine and spermidine compared to putrescine and cadaverine. These values are of the same order and close to the values of * K*_a_ obtained from isothermal titration calorimetry studies. Furthermore, thermal stabilization data also lend additional evidence to the AT specific binding of polyamines and the higher binding of spermine over spermidine to the DNAs.

### Ethidium Bromide Displacement Studies

**Figure 7:**
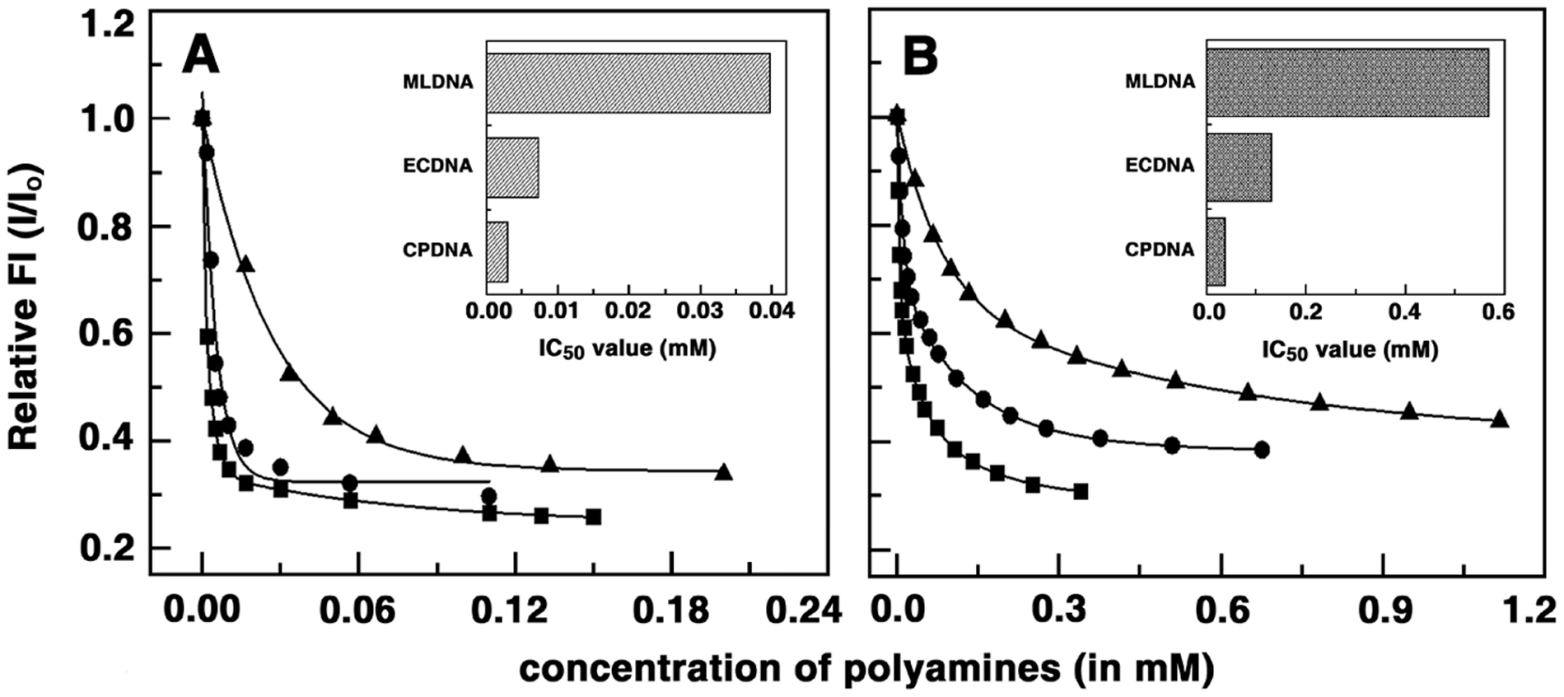
Displacement plots of ethidium bromide-DNA complexes by polyamines. Relative fluorescence intensity decrease of ethidium bromide (1.2 μM)-DNA(12.0 μM) complex induced by the binding of (A) spermine with CP DNA(-▪-), EC DNA (-•-), ML DNA (-▴-) and (B) spermidine with CP DNA(-▪-), EC DNA (-•-), ML DNA (-▴-) conducted in 10 mM SHE buffer pH 7.0 at 293.15 K (Inset: The values of IC_50_ of CP, EC and ML DNA shown as a bar graph).

As a sequel to the thermodynamic characterization of the association of the polyamines with different DNAs we examined their ability to displace bound ethidium bromide. Polyamines are not intercalating agents, but because of the presence of positive charge they bind to DNA possibly by strong electrostatic interaction. Polyamines can displace ethidium as reported earlier [36] with calf thymus DNA and this was probed for each of the polyamines with the DNAs. The results are graphically represented in Fig. 7. Ethidium bromide binds DNA through classical intercalation mode. The subsequent addition of polyamines to ethidium bromide-DNA complex allows the interaction of polyamines with the DNA backbone through electrostatic interaction with the negatively charged phosphate groups. This interaction leads to changes and modification in the helical nature of DNA thereby distorting the intercalation sites. Thus, the ethidium bromide molecule slips out of the intercalation site as a result of which the fluorescence decreases. Thus, addition of polyamines into an incubated solution of DNA-ethidium bromide induces displacement of ethidium bromide from the complex which leads to a decrease in the fluorescence of the complex. From the results it can be seen that the highest fluorescence decrease at low molar ratio was for spermine followed closely by spermidine for all the three DNA samples. The fluorescence quenching was much slow for putrescine and cadaverine. The largest ethidium bromide displacement was observed with CP DNA closely followed by EC DNA and then ML DNA. For all the three cases the maximum displacement was observed for spermine>spermidine >putrescine>cadaverine. From the fluorescence data the corresponding IC50 values were obtained which denote the concentration required to decrease the fluorescence intensity by 50%. A high value of IC_50_ indicates a very low ability to displace ethidium bromide from DNA. The IC_50_ values of spermine, and spermidine binding to CP DNA, were 2.2, 49.3 µM, respectively while that to EC DNA were 4.12, 72.3 µM, respectively. IC_50_ values of spermine, spermidine binding to ML DNA were 10.14, 200.5 µM, respectively. Thus, we can infer that the ability to displace DNA bound ethidium bromide was dependent on the charge as well as the molecular length; spermine having the highest length and maximum charge displaced much easier than cadaverine the smallest with lowest charge of the four. Also the IC_50_ values reflects the binding affinity of polyamines to CP DNA to be the strongest followed by EC DNA and then ML DNA. Therefore from this experiment also we can firmly conclude the clear AT base specificity of the polyamines.

### Characterization of Conformational Changes in DNA Induced by Polyamines

**Figure 8:**
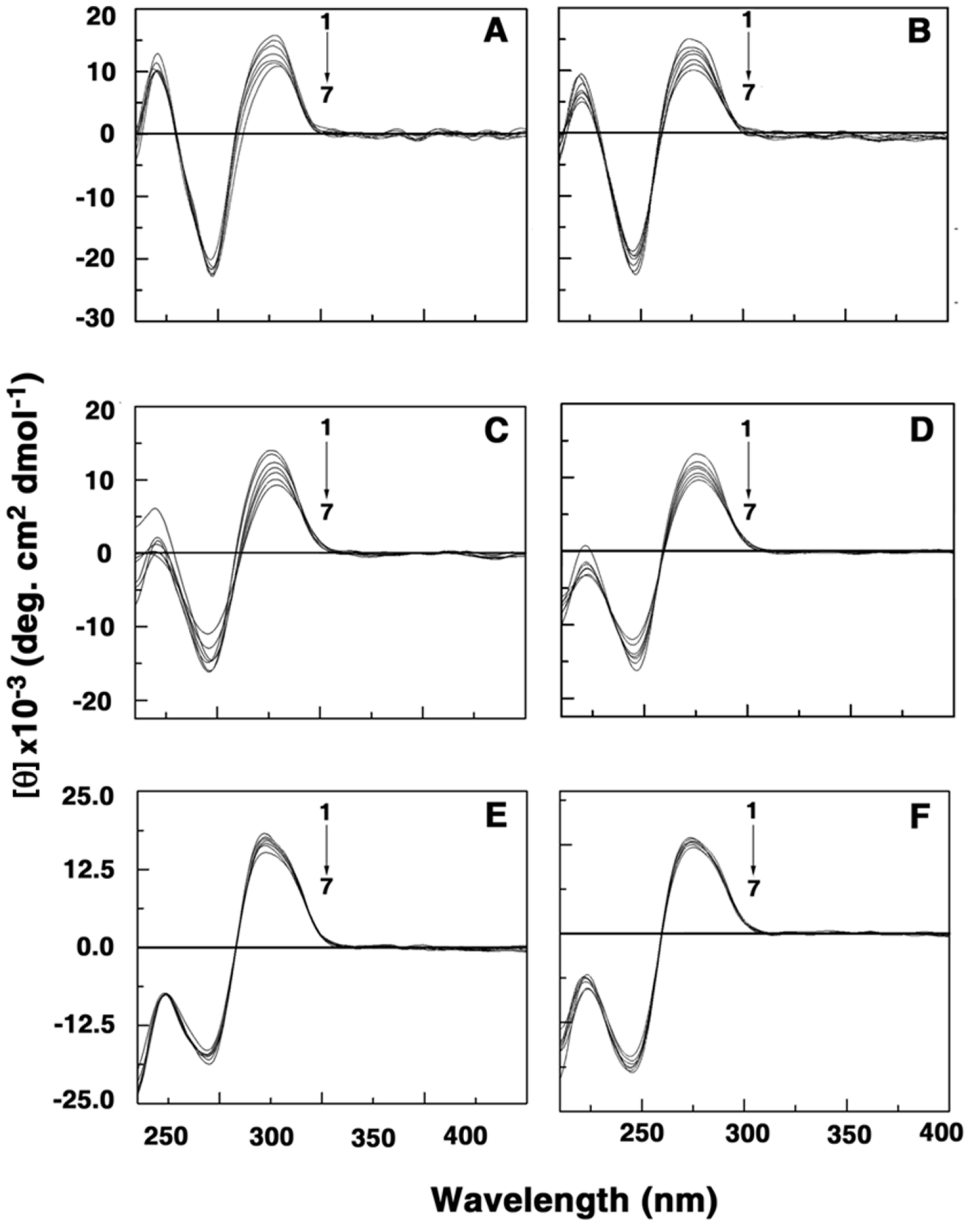
Circular dichroism spectral titration of DNA-poyamine complexes. Intrinsic CD spectra of (A) CP DNA (30 µM) treated with 0–63 µM (curves 1 to 7) spermine (B) CP DNA (30 µM) treated with 0–135 µM (curves 1 to 7 ) spermidine (C) EC DNA (30 µM) treated with 0–95 µM (curves 1 to 7) spermine (D) EC DNA (30 µM) treated with 0–175 µM (curves 1 to 7) spermidine (E) ML DNA (30 µM) treated with 0–120 µM (curves 1 to 7) spermine and (F) ML DNA (30 µM) treated with 0–250 µM (curves 1 to 7 ) spermidine at 293.15 K.

The above described results provided us the data on the binding and thermodynamic aspects of the interaction. The binding of polyamines and some analogs to DNA is reported to induce conformational changes in the DNA [25], [37], [61]. But a previous Raman spectral study suggested negligible effect on the B-form structure [36]. We now tested the comparative effects of these polyamines on the three DNAs by circular dichroism experiments. CD spectra at various D/P (polyamine/DNA base pair molar ratio) values were recorded in the 210–400 nm regions and are presented in Fig. 8. The intrinsic CD spectra of the B-form duplexes were characterized by a large positive band in the 270–280 nm and a negative band at 248 nm. There were, however, small differences in the ellipticity and wavelength maxima (Spectrum 1 of Fig. 8). These CD bands arise due to the stacking interactions between the base pairs and the helical structure that provide asymmetric environment for the nucleic acid bases [62]. Polyamines are CD inactive and hence the CD changes if any can be attributed to structural changes in the DNA resulting from the interaction. The binding of polyamines led to a decrease in the ellipticity of the positive band with a slight red shift in the wavelength maximum and a decrease of the negative band of the DNAs. This slight red shift is most pronounced for spermine and least for putrescine and cadaverine.Overall, the change was greater with CP DNA and least with ML DNA while on the other hand the change was more with spermine and lowest with cadaverine. Similar CD signal changes indicate similar type of interaction of polyamines with each of the three DNAs but the differences in the intensities indicated that the binding was sensitive to the base composition, being higher with the AT rich CP DNA followed by EC DNA and lowest with GC rich ML DNA. Thus, we can infer that the conformational changes within the B-form in each of the DNA duplexes due to the binding of these polyamine appear to be stronger with spermine and spermidine and that the CD results also confirm the base specificity of these polyamines whereby the preference of spermine over spermidine is followed by putrescine and cadaverine, in agreement with the results from calorimetric, fluorescence displacement and melting experiments.

### Conclusions

In this report the base pair specificity of four biogenic polyamines with three genomic DNA has been investigated. Our studies result in two major conclusions viz. the polyamines bound stronger with AT rich *Clostridium perfringenes* DNA and least with *Micrococcus lysodeikticus* DNA and the binding to each DNA was stronger for spermine and varied as spermine>spermidine >putrescine>cadaverine. Additionally, it has been revealed that the binding was influenced by salt concentration suggesting the contribution from electrostatic forces to the Gibbs energy of binding to be the dominant contributor. Parsing of the free energy of the binding also showed a major contribution from electrostatic forces in each case. The temperature dependent studies conferred that the binding was favoured by large positive entropy changes and decrease in enthalpy changes though to different expanse. Thermodynamics of the interaction revealed that the binding was entropy driven with small enthalpy contribution at all temperatures studied. Each system studied exhibited enthalpy-entropy compensation. The negative heat capacity changes in all systems studied are associated with the role of some hydrophobic interactions which arise due to the non polar interactions between DNA and polyamines. Binding of polyamines lead to thermal stabilization of each of the DNAs to different extents where again stabilization by spermine was maximum with CP DNA. Ethidium bromide displacement studies confirmed that spermine and spermidine displaced ethidium bromide from ethidium bromide-DNA complexes at a much lower concentrations compared to putrescine and cadaverine in each case. The binding to the DNAs resulted in significant perturbation of the conformation of each of the DNA duplexes where the most predominant effect was shown by spermine and least by putrescine and cadaverine in all three cases. Additionally the experiments indicate that the number of positive charges and molecular length of the polyamines have a profound effect on the complexation. Thus binding of spermine>spermidine >putrescine>cadaverine. These results may help us to engineer polyamine analogues that will affect cell function and inhibit growth in cancerous cells but will not serve the normal functions and produce changes in DNA as caused by natural polyamines.
